# Analysis of Immune-Cell Distribution of Bone Marrow in Patients with Myelodysplastic Syndrome

**DOI:** 10.3390/hematolrep15010005

**Published:** 2023-01-12

**Authors:** Chun-Liang Lin, Ching-Chan Lin, Tzu-Ting Chen, Wen-Jyi Lo, Shu-Ling Tzeng

**Affiliations:** 1 Institute of Medicine, Chung Shan Medical University, Taichung 40201, Taiwan; 2 Department of Internal Medicine, Taichung Hospital, Ministry of Health and Welfare, Taichung 40343, Taiwan; 3 Department of Hematology and Oncology, China Medical University Hospital, Taichung 404332, Taiwan

**Keywords:** apoptosis, lymphocytes, T cells, myelodysplastic syndrome

## Abstract

Myelodysplastic syndrome (MDS) immunity plays an important role in the proliferation and apoptosis of aberrant cells. Immune dysregulation has been studied in various prognostic subgroups. This study analyzed 60 patients with MDS via multidimensional flow cytometry to evaluate the expression of aberrant markers, such as CD7 and cytoplasmic CD3 on lymphocytes. The Revised International Prognostic Scoring System (IPSS-R) scores were used to classify the patients into risk groups. The results showed a significant downregulation of CyCD3− T cells in low–intermediate versus high-risk patients (*p* = 0.013). This study is the first to show that a significant decrease in cyCD3− T cells in patients with a lower IPSS-R score may indicate microenvironmental changes conducive to transformation in MDS.

## 1. Introduction

Myelodysplastic syndrome (MDS) describes a heterogeneous group of clonal hematopoietic disorders common in the elderly. These disorders are characterized by peripheral blood cytopenia and dysplastic changes due to ineffective hematopoiesis, recurrent cytogenetic abnormalities, and an increased risk of progression to acute myeloid leukemia (AML) [1].

Both genetic and epigenetic alterations, as well as immune mechanisms, may contribute to the pathologic mechanism of MDS, and these changes are the basis for the treatment plan and drug design [2]. For example, targeted therapies against epigenesis, such as azacytidine and decitabine, are available for the treatment of MDS. Nevertheless, the treatment of patients with MDS primarily depends on various risk-stratification tools, such as the International Prognostic Scoring System (IPSS) and its revised version (IPSS-R) based on the cytopenia, the percentage of blasts, and the cytogenetics [3]. Although some studies have confirmed that immunosuppressive therapy may lead to lasting hematologic responses and the abrogation of T-cell clones, particularly in low-risk MDS [4], applying this knowledge in routine clinical practice is difficult because of the lack of useful immune markers to stratify this heterogeneous disease.

Generally, MDS is believed to arise from a defect in stem cells. However, MDS clones are closely related to aberrant bone marrow (BM) stromal cells, which can even be the first event to cause MDS [5]. The BM is the site of hematopoiesis and has a varied population of cells, ranging from stem cells to almost-mature cells of each lineage, including erythrocytes, megakaryocytes, myelomonocytic cells (monocyte/macrophage and granulocytes), B cells, and natural killer (NK) cells [6].

Many studies have highlighted the role of different immune cells in the immune dysregulation leading to the pathogenesis of MDS and the disease’s progression to AML [6,7]. These factors include aberrant cytokine levels, increased T-helper cells and cytotoxic cells, a lower number of regulatory T cells, and dysfunctional NK cells, among others. Some studies also showed differences in the immune abnormalities of peripheral blood between patients who are at low risk and those at high risk for MDS [8,9]. Recently, a clinical trial aiming to rejuvenate immune cells by using immune-checkpoint inhibitors, such as nivolumab and ipilimumab, was conducted [10]. The discouraging results in MDS showed that there is still a significant knowledge gap that needs to be filled. Although the bone marrow can be viewed as a major organ, where hematopoietic cells interact with immune cells, the distinction between BM immune cells remains largely unclear. Thus, we designed this study to investigate the characteristics of BM immune cells between low- and high-risk MDS patients.

## 2. Materials and Methods

### 2.1. Study Population and Design

This retrospective study enrolled 60 patients with MDS who were followed in the Hematology Department of China Medical University Hospital from June 2017 to June 2020. Clinical and laboratory data were retrospectively collected from hospital records. Patients were considered to have anemia, neutropenia, and thrombocytopenia if their laboratory values were hemoglobin <12.5 g/dL, neutrophils <2.000 × 10^6^/L, and platelets <150 × 10^9^/L, respectively. The diagnosis and classification of MDS were established based on the World Health Organization criteria, revised in 2016 [11,12], after excluding other conditions that may contribute to BM dysplasia and/or cytopenia. All patients had an initial routine morphology assessment, conducted by an experienced hematopathologist. The IPSS-R score was calculated for all patients with MDS, as previously described [11]. The IPSS criteria were used to derive the karyotype-based risk classification [12]. 

### 2.2. Flow-Cytometry Studies

Flow cytometry (FC) was performed on BM samples from all patients at diagnosis, using an A LOT panel, as described by the Euroflow Consortium [13]. Briefly, samples were analyzed using eight antibodies in one tube. The following antibodies were used in this study: CyCD3-Pacific Blue (UCHT1, BD Biosciences, Jose, CA, USA), CD45-KrO (J.33, BeckmanCoulter, Brea, CA, USA), CyMPO-FITC (2C7, Cytognos, Salamanca, Spain), CyCD79a-PE (HM57, Cytognos), CD34-PerCP-cyanin5.5 (8G12, BD Biosciences), CD19-PE-cyanin7 (J3-119, Beckman-Coulter), CD7-APC (124-1D1, eBioscience, San Diego, CA, USA), and SmCD3-APC-C750 (SK7, BD Biosciences).

Acquisition of FC data was performed on an FACS Canto (BD Biosciences). Data were analyzed using Infinicyt software (Cytognos, Salamanca, Spain). The FC evaluation was performed in a randomized and blinded manner, with no knowledge of the mutational status and/or pathologic evaluation of the samples. For the evaluation of expression of aberrant markers, such as CD7 and cytoplasmic CD3, on lymphocytes, we superimposed the gated populations on positive- and negative-control populations, allowing control of intersample variations in fluorescence intensity. A mature T-cell population was defined as both surface-CD3-positive and cytoplasmic-CD3-positive cells; cyCD3-negative T cells were defined as a cluster of cells with surface CD3 positive but apparently dim cyCD3 expression [13].

We examined the CD34-positive/total nuclear-cell percentage (%), B-precursor/total nuclear-cell percentage (%), T-cell/total nuclear-cell percentage (%), B-cell/total nuclear-cell percentage (%), NK-cell/total nuclear-cell percentage (%), cyCD3-negative-T-cell/total T-cell percentage (%), CD7-negative-T-cell/total T-cell percentage (%), CD7-positive-NK-cell/total-NK-cell percentage (%), and CD7-positives among CD34-positive-cell/total CD34-positive-cell percentage (%).

### 2.3. Statistical Analysis

Statistical analyses were performed using the statistical program EZR (version 3.4.0). For comparisons of numerical variables, we used the Mann–Whitney U test, and we used the Pearson chi-squared test or Fisher’s exact test for comparisons of categorical variables. The cutoff for statistical significance was set at 0.05 (*p* < 0.05).

## 3. Results

### 3.1. General Characteristics and FC Data

A total of 60 consecutive patients with primary MDS were analyzed. Table 1 describes the general patient characteristics. The median follow-up time was 4.5 months (0.3–29 months). The sex distribution was 26 women and 34 men. The median age was 71 years (37–91 years). Of the 60 patients, 47 (78.3%) were transfusion-dependent and 32 (53.3%) had at least one infection within 3 months after diagnosis. Treatment with azacytidine was administered to 38 patients (63.3%). By the end of the follow-up, 34 (56.7%) patients had died.

Table 2 describes the distribution of the BM cells. Compared with the reference data from patients with normal or reactive BM [14], this cohort showed increased CD34+ myeloblasts (mean, 9.8% of total nucleated cells (0.03–69.3%)), decreased B precursors (mean, 0.06% of total nucleated cells (0.00–0.39%)) and mature B cells (mean, 0.79% of total nucleated cells (0.02–6.7%)), and decreased T cells (mean, 5.4% (0.08–23.2%)).

### 3.2. Immune-Cell Analysis of High- and Low–Intermediate-Risk Patients

Table 3 presents the results of the analysis of the BM blood cells at the time of initial diagnosis for the high-risk and low–intermediate-risk patients. The high-risk patients tended to show a higher percentage of CD34+ myeloblasts than the low–intermediate-risk patients (CD34+ cells/TNC (%)), presented as mean ± standard deviation (13.2 ± 13.7 vs. 6.5 ± 15.4; *p* = 0.079). No between-group differences were observed between the frequency of CD34+ B cells, mature B cells, NK cells, and T cells. However, the low–intermediate-risk patients had a higher percentage of cytoplasmic CD3− T cells than the high-risk patients (CyCD3− T cells/T cells (%); 6.49 ± 10.2 vs. 16.7 ± 19.1; *p* = 0.013). No statistical difference was found in terms of CD7− T cells, CD7+ NK cells, or CD7+ CD34+ myeloblasts between the high- and low–intermediate-risk patients.

## 4. Discussion

The results of this retrospective study showed differences in the distribution of immune cells in the BM between low–intermediate- and high-risk patients with MDS. A higher percentage of T cells with cytoplasmic CD3 downregulation was noted in the low–intermediate-risk than in the high-risk patients with MDS.

As the site of hematopoiesis, BM contains various precursor and differentiated cells, including myeloid, monocytic, erythroid, and B cells. By contrast, T cells develop in the thymus and are present in BM only with mature cells. Myelodysplastic syndrome is characterized by ineffective hematopoiesis, which produces aberrant clones prone to destruction through spontaneous apoptosis or immunosurveillance. However, increasing evidence has shown T-cell dysregulation in patients with MDS. The manifestation of autoimmune diseases has also been noted in these patients, particularly in low-risk patients. Additionally, interferon-gamma (IFNγ)-producing CD4+ T cells were demonstrated in BM in patients with MDS [15]. Moreover, proliferative CD8+ T cells specific for trisomy 8-related aneuploid hematopoietic progenitor cells have been observed [16]. Finally, BM T cells revealed a distinct functional signature with an elevated expression of IL-6, TNF-α, CCL4, CCL3, and IL-1RA [17]. Therefore, MDS is not only a disease of myeloid series but is also closely associated with T-cell dysregulation. Aberrant CD7, CD5, cCD79a, and cCD3 were found in 45.8%, 33.3%, 8.3%, and 8.3% of patients with acute myeloid leukemia, respectively [18].

Our study noted that the cytoplasmic CD3 expression differed between the low–intermediate- and high-risk patients with MDS. With its involvement in the regulation of the activation of both cytotoxic T cells and T helper cells, CD3 is a protein complex and T-cell co-receptor composed of four distinct chains, CD3γ, CD3δ, CD3ε, and CD3𝜁 [19]. The downregulation of CD3𝜁 has been reported in numerous pathologies and conditions associated with chronic inflammation; this chain can sense the sustained exposure to chronic inflammatory immune responses to restrict the magnitude of T-cell responses [20,21]. Dumont et al. showed that ZAP-70, an essential factor for T-cell activation, can control CD3 degradation to prevent T-cell hyper-responsiveness [22]. Although the pathogenesis of cytoplasmic CD3 downregulation in MDS is unclear, all these studies suggested that CD3 downregulation may be a manifestation of T-cell exhaustion. The higher percentage of cytoplasmic CD3 downregulation in lower-risk MDS patients may imply a more important role of immune dysregulation in low-risk than in high-risk MDS patients. Therefore a new strategy of CD3 T-cell–dependent bispecific (TDB) full-length humanized IgG1 therapeutic antibody targeting CLL-1 could potentially be used in humans to treat AML [23]. Whether CD3 downregulation can be used as a biomarker to predict immune response requires further investigation. 

The limitations of our study include the inherent unknown bias that may appear in any studies that are not prospective, randomized, or controlled. Second, there was selection bias in the convenience sequential sampling and the lack of control for medical conditions. Third, the clinical significance of cytoplasmic CD3 expression in MDS was not observed because of the small sample size of this study. Additionally, no significant aberrancy in other subsets, such as B cells and NK cells, was noted, except the counts of the CD34-positive cells. Considering the increasing diversity between different immune cells, it is obvious that our current panel cannot capture all the differences between the immune cells in MDS patients. 

Hence, this study and its results should be viewed as a hypothesis-forming analysis, and further prospective studies are necessary.

## 5. Conclusions

To conclude, our study showed different immune-cell distributions between low–intermediate- and high-risk MDS patients. Specifically, the increased downregulation of cytoplasmic CD3 expression was noted in the low–intermediate-risk MDS patients. The clinical significance and pathogenesis must be explored further.

## Figures and Tables

**Table 1:** Baseline clinical characteristics of 60 patients.

Clinical Characteristics	Number	%
Age (median, range)		71, 37–91
Sex, male	34	56.7
Risk groups (IPSS-R > 4.5)	30	50.0
Transfusion-dependent	47	78.3
Treatment with azacitidine	38	63.3
Mortality at the end of follow-up	34	56.7
ANC (leukocyte) score < 0.8	19	31.7
≥0.8	41	68.3
Hemoglobin score < 8	34	56.7
8~<10	18	30.0
≥10	8	13.3
Platelets (thrombocytes) score <50	28	46.7
50~<100	13	21.7
≥100	19	31.6
Cytogenetics Very good	1	1.6
Good	30	50.0
Low	10	16.7
Poor	4	6.7
Very poor	15	25.0
BM Blast % score ≤ 2	27	45.0
>2~< 5%	3	5.0
5~10%	3	5.0
>10%	27	45.0

IPSS-R = Revised International Prognostic Scoring System.

**Table 2:** Distribution of bone-marrow cells.

Cell	Mean	Median	Range	Reference Range
CD34+/TNC, %	9.8	3.8	0.03–69.3	0.27–1.6
B precursors/TNC, %	0.14	0	0–4.71	0.11–1.0
T cells/TNC, %	5.4	4.5	0.08–23.2	5.7–21
B cells/TNC, %	0.79	0.37	0.02–6.7	1.4–8.6
NK cells/TNC, %	2.39	1.28	0.01–15.9	0.42–5.3
cyCD3-negative T cells/T cells	11.6	3.6	0–61.9	NA
cyCD3-positive T cells/T cells	88.4	96.4	38–100	NA
CD7-negative T cells/T cells, %	11.9	7.7	0–86.5	NA
CD7-positive NK cells/NK cells, %	65.5	80.5	0.18–100	NA
CD7+positive CD34+ cells/CD34+ cells, %	13.9	4.9	0–91.5	NA

TNC = Total nucleated cell; NA = Not applicable.

**Table 3:** Bone-marrow analysis of high-risk and low–intermediate-risk patients.

	High Risk			Low–Intermediate Risk	
Flow Cytometry Data	*n*	(Mean ± SD)	*n*		*p* Value
CD34+/total TNC (%)	30	(13.2 ± 13.7)	30	(6.5 ± 15.4)	0.079
B precursor/TNC (%)	30	(0.10 ± 0.13)	30	(0.19 ± 0.86)	0.570
T cell/TNC (%)	30	(5.3 ± 4.5)	30	(5.5 ± 5.5)	0.897
B cell/TNC (%)	30	(0.92 ± 1.36)	30	(0.66 ± 0.89)	0.396
NK cell/TNC (%)	30	(2.37 ± 3.14)	30	(2.40 ± 3.03)	0.961
cyCD3-T/T (%)	30	(6.49 ± 10.2)	30	(16.7 ± 19.1)	0.013
CD7-T/T (%)	30	(9.64 ± 10.9)	30	(14.1 ± 21.3)	0.319
CD7+NK/NK (%)	30	(67.1 ± 30.9)	30	(63.8 ± 35.2)	0.700
CD7+CD34+/CD34 (%)	30	(15.9 ± 23.5)	30	(11.9 ± 17.6)	0.453

(X^2^ squire test) (independent -sample *t*-test). High-risk patients tended to show a higher percentage of CD34+ myeloblasts than the low–intermediate-risk patients. Low-risk patients had a higher percentage of cytoplasmic CD3− T cells than high-risk patients, *p* = 0.013.

## Data Availability

The datasets generated and analyzed in the current study are available from the corresponding author upon reasonable request.
